# Liposomal delivery system/adjuvant for tuberculosis vaccine

**DOI:** 10.1002/iid3.867

**Published:** 2023-06-09

**Authors:** Melika Moradi, Farzaneh Vahedi, Arian Abbassioun, Arash Ramezanpour Shahi, Mohammad Sholeh, Mortaza Taheri‐Anganeh, Zahra Dargahi, Roya Ghanavati, Seyyed Hossein Khatami, Ahmad Movahedpour

**Affiliations:** ^1^ Department of Microbiology, School of Medicine Ahvaz Jundishapur University of Medical Sciences Ahvaz Iran; ^2^ Department of Medical Biotechnology, School of Advanced Medical Sciences and Technologies Shiraz University of Medical Sciences Shiraz Iran; ^3^ Department of Virology, Faculty of Veterinary Medicene University of Tehran Tehran Iran; ^4^ Department of Veterinary Clinical Sciences, Poultry diseases and hygiene Resident, Faculty of Veterinary Medicine Shahrekord University Shahrekord Iran; ^5^ Department of Bacteriology Pasteur Institute of Iran Tehran Iran; ^6^ Cellular and Molecular Research Center, Cellular and Molecular Medicine Research Institute Urmia University of Medical Sciences Urmia Iran; ^7^ Behbahan Faculty of Medical Sciences Behbahan Iran; ^8^ Department of Clinical Biochemistry, School of Medicine Shahid Beheshti University of Medical Sciences Tehran Iran

**Keywords:** *Mycobacterium tuberculosis*, subunit vaccine, vaccination

## Abstract

As reported by the World Health Organization, about 10 million individuals were infected with tuberculosis (TB) worldwide. Moreover, approximately 1.5 million people died of TB, of which 214,000 were infected with HIV simultaneously. Due to the high infection rate, the need for effective TB vaccination is highly felt. Until now, various methodologies have been proposed for the development of a protein subunit vaccine for TB. These vaccines have shown higher protection than other vaccines, particularly the Bacillus culture vaccine. The delivery system and safety regulator are common characteristics of effective adjuvants in TB vaccines and the clinical trial stage. The present study investigates the current state of TB adjuvant research focusing on the liposomal adjuvant system. Based on our findings, the liposomal system is a safe and efficient adjuvant from nanosize to microsize for vaccinations against TB, other intracellular infections, and malignancies. Clinical studies can provide valuable feedback for developing novel TB adjuvants, which ultimately enhance the impact of adjuvants on next‐generation TB vaccines.

## Graphical abstract

The delivery system and safety regulator are common characteristics of effective adjuvants in tuberculosis (TB) vaccines and the clinical trial stage. Based on our findings, the liposomal system is a superb adjuvant for vaccinations against TB, other intracellular infections, and malignancies.

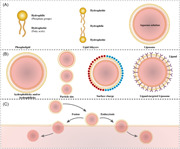

## 1 INTRODUCTION

Tuberculosis (TB) is an infectious disease and a intracellular pathogen caused by *Mycobacterium tuberculosis* (*M. tb*) bacteria or *Mycobacterium bovis* (*M. bovis*).^1^, ^2^ While chemotherapy and vaccines are mostly applied to combat TB, this bacterial infection is still among the most dangerous infectious diseases and affects one‐third of the population in the world.^3^ Unfortunately, the majority of TB patients have no symptoms while carrying *M. tb* during their lifetime.^4^ In spite of the dramatic reduction in the mortality and prevalence of TB in recent years, it is the second leading global cause of death from communicable diseases.^5^ Recent research has reported about 10 million new cases of TB, of which almost 1.5 million died of TB. Among those who died, 214,000 cases were infected with HIV. The incidence of TB has been demonstrated to be severe in Southeast Asia with 43% of new cases, followed by Africa and the Western Pacific region with 25% and 18% of new cases, respectively.^6^, ^7^

Owing to the presence of bacillus TB in more than 25% of the world's population, multidrug resistance and extensive drug resistance are growing among *M. tb* strains.^8^ Increased mortality from TB/HIV coinfection makes the risk of TB more dangerous to public health; thus, the search for a vaccination that could inhibit the disease from spreading is of paramount importance.^9^, ^10^ The first‐line drugs (isoniazid, ethambutol, rifampicin, and pyrazinamide for the treatment of TB are presently tolerated by Mycobacterium, giving rise to the resistance of bacteria to multiple drugs.^11^ The efficient immune mechanisms that make susceptibility to infection and disease are not yet comprehensively realized. With the goal of terminating the epidemic of TB and decreasing the prevalence and mortality of this disease, the World Health Organization has put extensive efforts into precisely diagnosing and developing a new therapeutic vaccine for the disease.^12^ Notwithstanding broad signs of progress in pharmacological and diagnostic experiments, the only cost‐effective choice for long‐term control of this infectious disease is via effective immunization.^13^

Vaccines induce cellular immune, T helper 1 (Th1), and cytotoxic T lymphocytes (CTLs) against infections living inside cells, for example, *M. tb*.^14^ It has been well established that CD4+ T cells play a vital role in cellular immunogenicity. Th1 cells (CD4+) are also necessary for combating intracellular pathogens such as *M. tb*. Th1 cells secrete cytokines such as interferon gamma (IFN‐γ) and Tumor necrosis factor‐α (TNF‐α) to invoke and interleukin‐2 (IL‐2) to activate T cells, as well as innate immune cells.^15^, ^16^ *Bacillus* Calmette Guérin (BCG) vaccine was first developed from the attenuated live strain of *M. Bovis* by Albert Calmette and Camille Guerin, two French scientists. This vaccine is known as one of the most significant strategies to control the disease for a long time.^17^ Randomized trials have estimated from 80% to no protection for BCG vaccines; therefore, the capacity of BCG to protect against TB is controversial.^18^ Likewise, as BCG is a live attenuated vaccine, it is not suggested for individuals with weakened immune systems, such as HIV‐positive infants.^19^ Thus, it can be deduced that the TB vaccine should have the ability to inhibit new infections and completely eliminate *M. tb* infection over a long period of time.^20^ Attempts to replace BCG have been relatively satisfactory owing to the lack of understanding of immunity to *M. tb* infection. Although the anti‐*M. bovis* BCG is the only licensed and cost‐efficient vaccine against neonatal TB, particularly in teenagers and adults; it has been indicated to be ineffective in avoiding active disease.^21^ According to age structure modeling, vaccination for low‐ and middle‐income people has been found to have a higher effect on the TB burden worldwide. Moreover, it is more cost‐effective than vaccination for infants only, even if it is less effective or more expensive.^22^ Thus, the need for new vaccines has attracted much interest, and ongoing attempts have been made in this area. The main strategy for the development of virus vector vaccines, entire cells, or mycobacterial lysates and adjuvanted recombinant protein components could be the use of novel TB vaccines.^23^

At present, there are various TB vaccines that have undergone different phases of clinical trials (Table 1). These vaccines are generally categorized into three main groups. The first group includes live or attenuated recombinant BCG vaccines. These vaccines could be an alternative to the available BCG vaccine and have shown high immunogenicity and protection against the disease. The second group of vaccines entails viral vector vaccines and adjuvanted subunit vaccines. These non‐BCG candidate vaccines are used as boosters after the previous dose. The third group is vaccines derived from *Mycobacterium* whole cell or fragmented. These vaccines serve as a therapeutic vaccine or chemotherapy supplement to decrease the time it takes to treat active TB or latent TB infection.^24^, ^25^

**Table 1:** Potential tuberculosis vaccine candidates at the clinical trial stage.

Name of vaccine	Type of vaccine	Vaccine composition	Phase	Clinical trials gov. identifier
M72/AS01E	Subunit vaccine	Mtb32A and Mtb39A fusion protein with AS01E adjuvant.	IIb	NCT04556981
H56:IC31	Subunit vaccine	Ag85B, ESAT‐6, and latent Rv2660c fusion protein with IC31 adjuvant.	II	NCT03512249
ID93 + GLA‐SE	Subunit vaccine	Rv1813, Rv2608, Rv3619, Rv3620 fusion protein with GLA‐SE adjuvant	I	NCT03806699
GamTBvac	Subunit vaccine	Ag85A and ESAT6‐CFP10 fusion protein with dextran‐binding domain immobilized on dextran, DEAE‐dextran core adjuvant, and CpG oligodeoxynucleotides	III	NCT04975737
H4:IC31	Subunit vaccine	H4 antigen with IC31 adjuvant.	I	NCT02378207
AEC/BC02	Subunit vaccine	BC02 adjuvant with Ag85b antigen and ESAT‐6/CFP‐10	Ib	NCT04239313
TB/FLU01L	Recombinant live vaccine	Recombinant influenza virus with a replication defect A expressing the antigen ESAT‐6	I	NCT03017378
Ad5Ag85A	Recombinant live vaccine	Ag85A‐expressing Adenovirus serotype 5	I	NCT02337270
MVA85A	Recombinant live vaccine	Ankara, a recombinant Vaccinia virus with a replication defect that expresses Ag85A	IIb	NCT03681860
TB/FLU04L	Recombinant live vaccine	Antigens Ag85A and ESAT‐6 are expressed in an attenuated replication‐deficient influenza virus vector	I	NCT02501421
ChAdOx1‐ 85A	Recombinant live vaccine	Chimpanzee adenoviral expressing Ag85A	I	NCT03681860
VPM1002	Recombinant live vaccine	BCG recombinant vaccine containing the gene for listeriolysin O	III	NCT04351685
MTBVAC	Attenuated live vaccine	Clinical isolate of *Mycobacterium tuberculosis* with ESAT6 and CFP10, as well as independent stable genetic deletions of the phoP and fadD26 genes	III	NCT0497517
DAR‐901	Inactivated TB vaccine	SRL172 produced on agar using a scalable, broth‐grown production method	II	NCT02712424
RUTI	Inactivated TB vaccine	Detoxified, fragmented *M. tuberculosis* polyantigenic liposomal vaccine	II	NCT04919239
Vaccae	Inactivated TB vaccine	M. vaccae that has been killed by heat	III	NCT01979900
NCT01979900	DNA vaccine	*M. tuberculosis* antigen plasmids and Flt3 ligand	I	NCT03159975

## 2 APPROACHES TO SUBUNIT VACCINE

The COVID‐19 pandemic's progress and its unforeseen worldwide effects have brought attention to the urgent need for safe, dependable and effective vaccines.^26^, ^27^ The messenger RNA vaccine family is one of the cutting‐edge immunization classes that has produced exceptional success against infectious disorders during the past 10 years.^28^, ^29^ This type of vaccine offers a number of significant advantages over conventional platforms, including the ability for highly quick and flexible vaccine design and production.^30^, ^31^ Additionally, it has also been demonstrated that attenuated live vaccines are a highly effective method for preventing viral infections^32^, ^33^

**Figure 1:**
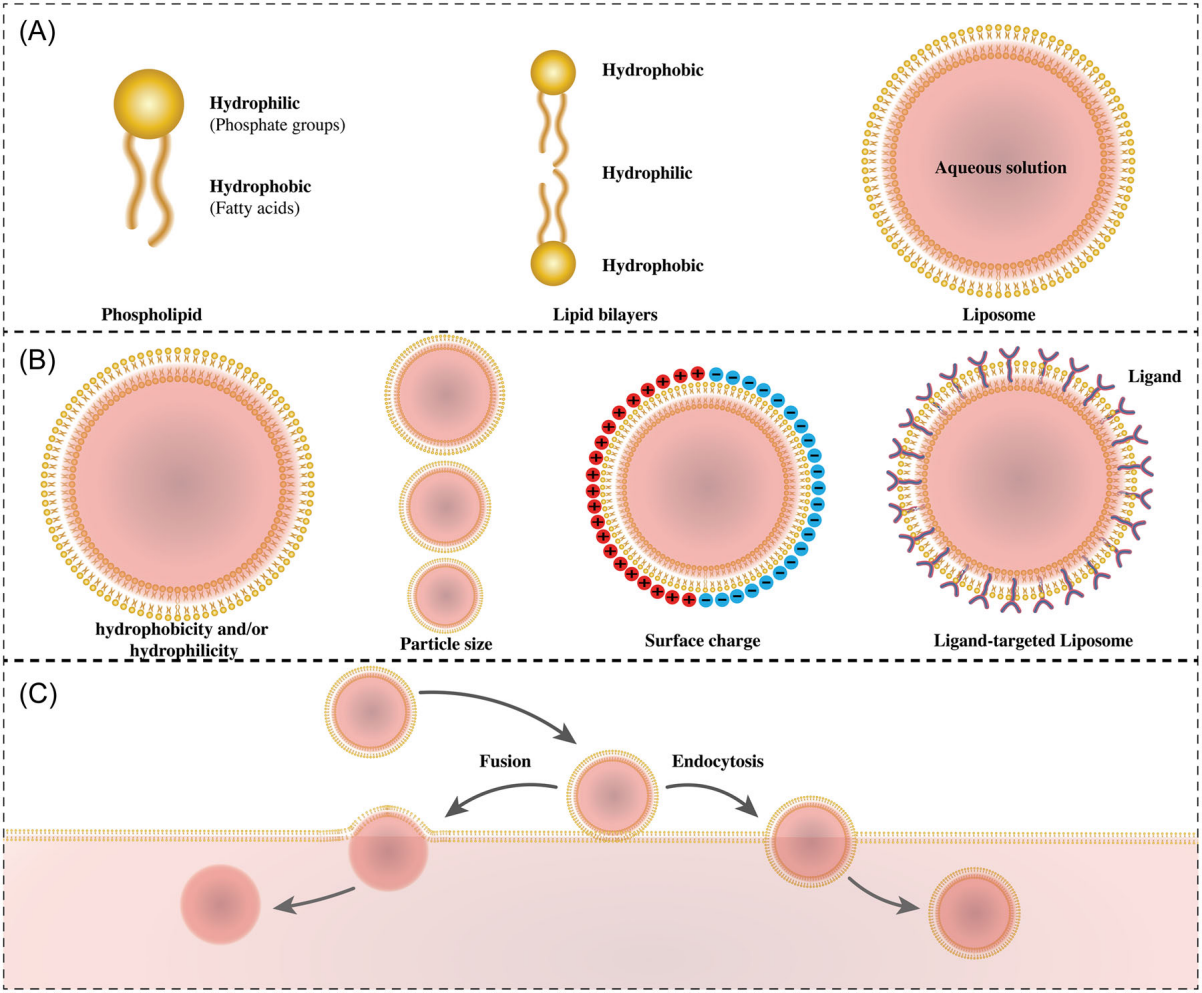
A schematic view of (A) a liposome structure (B) an antigen conjugated liposome (C) entering the liposome into a target cell is shown.

**Figure 2:**
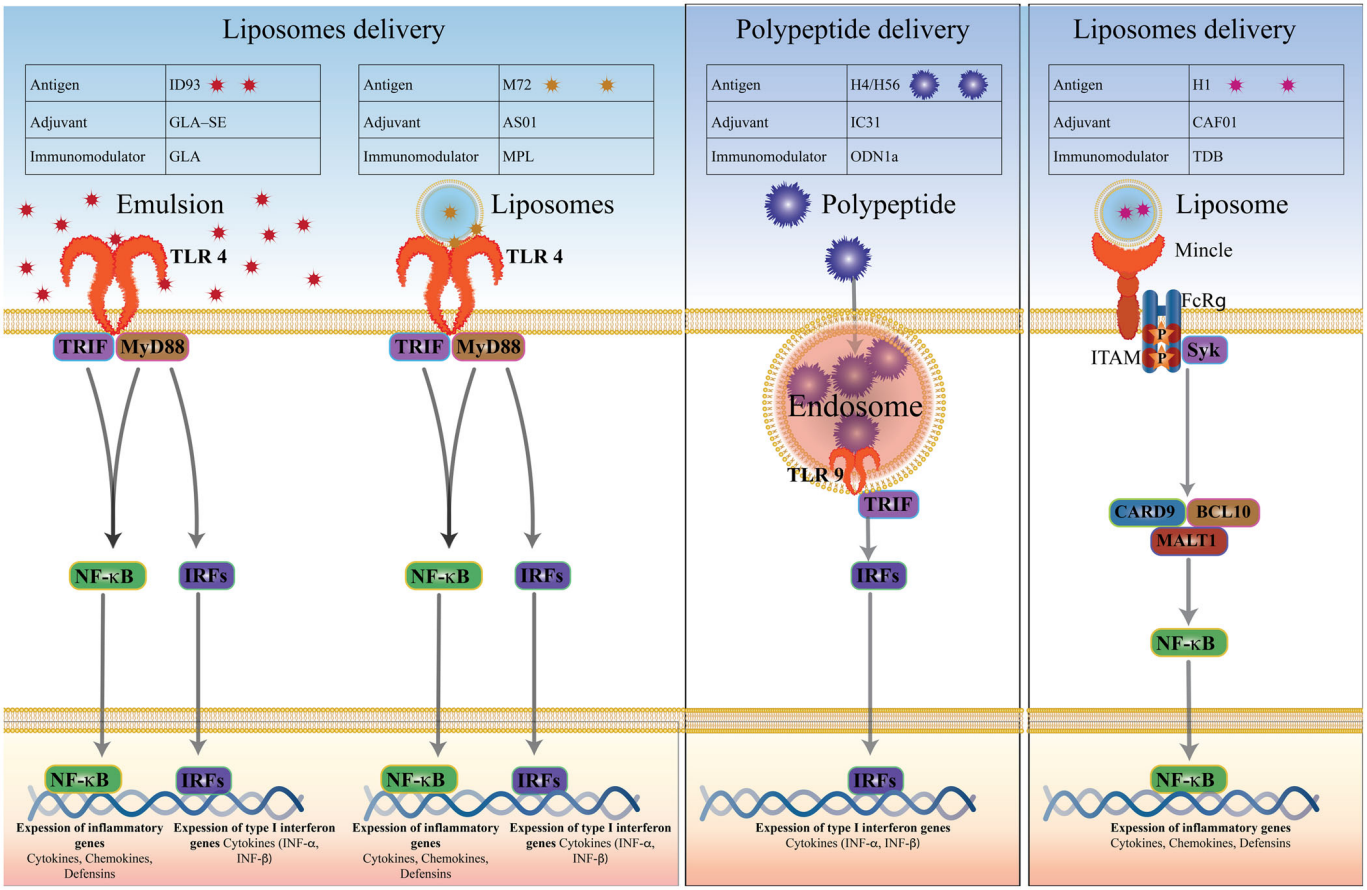
Types of antigen/adjuvant drug delivery systems used in the prevention treatment of TB. TB, tuberculosis.

Subunit vaccines have been introduced as a safer alternative to attenuated live vaccines.^34^ These vaccines comprise highly pure recombinant antigens, and this feature makes them have higher purity and lesser immunosuppressive components than traditional vaccines. Subunit vaccines do not contain pathogenic microorganisms, but they use pathogenic antigens; for these reasons, they are safer than inactivated vaccines.^35^ Sometimes, adjuvants are added to subunit vaccines to control the adaptive immune response and provide necessary innate immunopotentiation.^36^ Adjuvants are a heterogeneous category of chemicals serving as functional excipients. These agents are often categorized into two classifications based on their mechanism of action. Delivery systems act as carriers of antigens and immune stimuli in vaccines, often in the form of liposomal particles, emulsion droplets, or immune‐stimulating complexes.^37^, ^38^ immune potentiators, such as the ligands for the toll‐like receptor (TLR) (Figure 1).^39^ It has been shown to be needed for simultaneous antigen presentation and activation of antigen‐presenting cells (APCs).^40^ Liposomes, emulsions, mineral salts, and biodegradable polymers are routine transport systems, and the first two systems are currently applied as TB adjuvants (Figure 2).^41^ The selection of a proper adjuvant will not only increase the response level but also determine the immune response type.^42^

## 3 LIPOSOMAL ADJUVANTS FOR SUBUNIT VACCINE

Since 1974, liposomes have been introduced as adjuvants. Vaccination of mice with diphtheria toxin (DT) with liposomal adjuvant has been indicated to be immune to the DT disease and induce higher antibody titers compared to the vaccination of mice with non‐adjuvant DT.^43^ Using liposomal adjuvants has been assessed repetitively in clinical trials.^44^ These adjuvants may act as transport structures for subunit antigens and as immunopotentiators.^45^ Liposomal adjuvants are extremely adaptable as they can be altered by the lipid composition,^38^ adding immunostimulating substances,^38^ formulation techniques, antigen mode, and immunostimulatory relationship.^46^ Liposomes have a spherical structure and comprise one or more phospholipid layers. Their structure is very similar to cell membranes and includes different substances, for example, proteins and polysaccharide antigens.^47^

## 4 PHYSICOCHEMICAL PROPERTIES OF LIPOSOMAL ADJUVANTS

An alternative method for developing novel TB vaccines is using liposomes as an antigen carrier for subunit vaccinations. Liposome properties directly influence the immunological response to an antigen, which can be altered in terms of charge, composition, and size.^48^ For the first time, liposomes produced by the self‐assembly of certain amphiphilic lipids in an aqueous solution were used as model membranes. Amphiphilic lipids with a cylindrical shape have a propensity for generating lamellar phases, and when equilibrated with excess water, they may create closed vesicles, which are often formed of varied lipid bilayers separated by watery layers (multilamellar liposomes).^46^ The lipid composition of liposomes and their instruction technique affirm the chemical features (particle size, membrane liquidness, hydrophobicity, and surface charge) of vesicles. Therefore, the chemistry features of liposome dispersions can be managed through both their composition and instruction approach.^49^ The liposome composition may also influence the integration methods (immunostimulators and subunit antigens) applied to other molecules to validate the type and level of immune response generated by the vaccine.^50^ The adjuvant action of liposomes is defined by their ability to become involved in APCs and enhance the exposure of antigens and immunostimulators to APCs.^46^ When utilized as adjuvants, liposomes function as delivery structures for antigens and immunostimulants. Flexibility is one of liposome advantages that allows molecules to be combined in the same liposome dispersion, that is, a lipid‐based immunostimulator and a protein‐based antigen.

Liposomes greater than 225 nm elicit Th1 immunological responses.^51^ It has also been explored that the adjuvant type, but not the liposome size, has a role in controlling the immunological response elicited by vaccination formulations.^52^ Liposomes of varying sizes and lamellarity, when coupled with a protein antigen, have various abilities to trigger humoral and cellular immunity. Liposomes with a diameter of only ~600 nm would induce greater cellular and humoral adaptive immune responses than multilamellar vesicles with a diameter of two times larger.^53^ In terms of uptake by professional APCs, cationic liposomes perform better than neutral and anionic liposomes because proteins are restricted to the aqueous compartment of the liposomes.^14^ For the production of Th1 responses, the capacity of various liposomes in combination with GLA was tested. The result showed that anionic liposomes are efficient when Th1 responses increase.^14^ Some of the cationic liposomal adjuvants that progressed to human clinical trials are JVRS‐100, Vaxfectin, CAF01, and LPD.^54^ The main component of CAF01 comprises the cationic liposome dimethyl‐dioctadecyl ammonium (DDA); it contains the hydrophilic head group of dimethylammonium bonded to two hydrophobic 18‐carbon alkyl chains.^55^ DDA spontaneously separates into two vesicular layers in an aqueous medium and is used as a way of transferring antigens due to this feature.^56^ Since it has a net positive charge, it will readily attach itself to cells with a high concentration of negatively charged surface molecules. Moreover, DDA is also capable of binding negatively charged proteins and DNA molecules, thus introducing these antigens into APC cells.^57^ Also, by fusion with endosomal membranes or cross‐presentation, DDA can deliver antigens antigen to the cytosol. Afterward, it transports the generated peptides to the endoplasmic reticulum via a transporter that can process antigens to affect the response of CD8+ T cells against the protein antigen, which must be loaded on the MHC class I molecule for presentation to T‐cells.^58^ Studies on cationic liposomes demonstrated that the stiffness of the delivery mechanism, in addition to charge, is a significant factor in the development of antigen depots. A comparison of two liposome delivery systems indicated that the rigid DDA system could permanently maintain and slowly release both liposome and vaccine antigen from the injection site, which was satisfactory for sustained Th1 responses.^59^, ^60^ The two systems were different only in terms of the degree of acyl chain saturation, that is, providing DDA rigid and dioctadecyldimethylammonium fluid at physiological temperature.

## 5 LIPOSOMAL SUBUNIT VACCINES AGAINST TB

Older experimental TB adjuvants were considerably intricate preparations, with the consisting finding that mixing, for instance, a cell wall extract in liposomes or oil droplets resulted the best efficacy.^41^ In clinical studies, there are 12 TB vaccine candidates, of which eight of these vaccines are protein subunit vaccines.^61^ One obvious benefit of subunit vaccinations is their higher safety profile compared to live attenuated vaccines, which cannot always be administered to immunocompromised people.^62^ However, subunit vaccines need an adjuvant to evoke an important memory immune response to the vaccination antigen. In addition, there are no clinically approved adjuvants that elicit antigen‐specific effectors and long‐lived memory CD4+ and CD8+ T cells.^63^ Thus, for the development of a vaccine, it is necessary to find a novel adjuvant that could induce a well‐defined cell‐mediated immune response.^64^ AS01E, IC31, GLA‐SE, and CAF01 are adjuvants tested in clinical trials for use in TB subunit vaccines. The first adjuvant, AS01E, consists of monophosphoryl lipid (MPL) and *Quillaja saponaria* (QS21) as immunostimulants.^65^ IC31 adjuvant is a TLR9 agonist containing a cationic peptide (KLKL (5) KLK) and a synthetic oligodeoxynucleotide (ODN1a),^66^ GLA‐SE adjuvant is a stable squalene‐in‐water emulsion comprises of a synthetic TLR4 agonist GLA (SE).^67^ CAF01 is a liposomal adjuvant composed of DDA and TDB (trehalose‐6,6‐dibehenate), a synthetic analog of trehalose‐6,6’‐dimycolate (TDM) a component of mycobacterial cell walls (Table 2).^68^ All of the above‐mentioned adjuvants are complex formulations consisting of vehicles and an immunostimulator. It has been demonstrated that the quantity and quality of the.^69^ MPL and QS21, which are available in an oil‐in‐water emulsion (AS02) or liposomes (AS01), are at the heart of GSK's Adjuvant Systems.^70^ GSK antigen candidate M72 has been adjuvanted with AS01E. Several clinical trials have found that AS01E causes a very strong CD4+ T cell response, with both Th1 and Th2 cytokine combinations, as well as the activation of CD8+ T cells and NK cells without major adverse effects.^71^ TLR9 (and TLR3) agonists have been exhibited to be successful in eliciting robust CD8+ T cells. Liposomes complexed with the TLR7, TLR4, and TLR2 agonists were able to produce marginally stimulating responses in CD8+ T cells.^72^ When with The liposome TLR9‐agonist complex, namely LANAC, when paired with ESAT‐6, vaccination makes considerable protection; however, it is most likely that the protective effect is mediated by CD4+ T cells.^41^ DDA and MPL were used as a lipid in liposomes and an adjuvant in TB vaccinations, respectively.^73^

**Table 2:** The use of adjuvant systems in TB clinical trials.

Adjuvant	Antigen	Delivery	Immunomodulator	Signaling pathway
GLA–SE	ID93	Emulsion	Glucopyranosyl lipid adjuvant (GLA)	TLR4
AS01	M72	Liposomes	3‐O‐desacyl‐4′‐monophosphoryl lipid A (MPL)	TLR4
IC31®	H4/H56	Polypeptide	ODN1a stands for oligodeoxynucleotide.	TLR9
CAF01	H1	Liposomes	TDB; mycobacterial cord factor synthetic variation	Mincle

There is no doubt that liposomal components of mycobacterial lipids can induce potent humoral and cellular immune responses to both mycobacterial and nonmycobacterial antigens.^74^ The lipid extract of mycobacteria consists of various lipids separated by thin‐layer chromatography and immunostimulatory molecules. One advantage of using complicated instructions for vaccine administration is to activate many portions of the proinflammatory cascade, which leads to broader and longer‐lasting biological activity.^75^ In a study, liposomes based on phosphatidylinositol mannosides (PIMs) isolated from BCG were investigated as a probable antigen delivery mechanism. Human dendritic cells were stimulated by the PIMs, and animals immunized with ovalbumin emulsified in PIM liposomes developed ovalbumin‐specific antibodies and cytotoxic T‐cell responses.^76^ Another study utilized mycobacterial lipids on their own. A highly stronger immune response was induced when these lipids were combined with cationic liposomes.^77^ The cationic surfactant DDA, in comparison to other liposomes, was distinguished as the most efficient tool in terms of antibody production and also the amount of IFN‐γ induced. In spite of the use of DDA as an adjuvant for a long time,^59^ humans have been given the drug.^78^ However, its exact role as an adjuvant is still unclear. It has been proven that DDA is a very beneficial gene uptake facilitator in the transfection field. It is also speculated that DDA interacts with negatively charged cell membranes through its positive charge.^79^ Thus, it could be the same activity that allows DDA to boost antigen uptake and increase immunomodulatory mycobacterial lipids by cells presenting an antigen.^80^

Muramyl dipeptide (MDP) has mainly been utilized in vivo for cancer treatment purposes and has indicated anti‐influenza activity. However, it has limited and unsuccessful applications in TB vaccines. The traditional prophylactic aerosol challenge test did not provide protective effects when mice were administered MDP and DDA liposomes combined as an adjuvant for the *M. tb* antigen ESAT‐6.^73^, ^81^ In a previous investigation, the DMT‐liposome adjuvant component CTT3H was introduced as a potential candidate for a TB vaccine, though further preclinical and clinical testing was required. In another study, the adjuvant DMT was produced by combining MPL with TDB into a DDA liposome.^82^

The adjuvant MPL is a low toxic synthetic variant of lipopolysaccharides, the agonist of TLR‐4, that is used in the adjuvants AS01 and AS02. MPL has been approved for use in currently used HPV and HBV vaccines.^83^, ^84^ TDB is a synthetic analog of TDM that activates APCs through the Mincle receptor and FcRgamma‐Syk‐Card9 signaling, inducing significant Th1 and Th17 immunological responses in vaccinated mice.^84^, ^85^ CAF01 (TDB in liposome) has been highlighted to have a potent adjuvant impact on cellular and humoral responses against TB and HIV.^45^, ^86^ In vaccinated mice, DMT had the ability to elicit an antigen‐specific CD8+ T cell response. Moreover, DMT‐adjuvanted CTT3H, in comparison to the BCG group, induced more antigen‐specific IFN‐γ + or TNF‐α + CD8 + T cells, suggesting a larger CTL impact.^87^, ^88^

Mtb72F/AS02A is a subunit vaccine against TB and a fusion of the Mtb39a and Mtb32a *M. tb* antigens with the adjuvant AS02A; This vaccine mostly induces Th1 immune responses.^89^ Mtb72F/AS02A is currently being tested in phase II clinical trials to find if it can improve pre‐existing BCG immune responses.^90^, ^91^, ^92^, ^93^ M72/AS01 is a liposomal formulation of MPL A and QS‐21 and has been indicated to be safe. M72/AS01, compared to M72/AS02 and Mtb72F/AS01 vaccinations, induces greater cell‐mediated immunity in *M. tb*‐negative patients.^92^ Hybrid 1 (IC31) consists of antigens Ag85b and ESAT‐6 and is used in comparison to the adjuvant IC31 in cationic peptides containing CpG‐DNA.^89^ This vaccine targets antigens that *M. tb* does not express during its latent period, though they produce excellent immune responses.^94^

The use of liposomes in TB vaccine formulations has shown favorable results.^95^ Simple production and low toxicity, and immunogenicity are the advantages of liposomes. However, liposome formulations comprising LTB‐related antigens are not common. Liposome nanoparticle production seems to be a potential method for developing a vaccine, and cationic liposomes, such as CAF01, have been applied to treating TB.^96^ According to new research, specific Th1 and Th17 responses to H56/CAF01 vaccine‐induced subcutaneous immunization were maintained following spray drying of the vaccine. Moreover, spray drying did not change the physicochemical properties of CAF01 liposomes.^97^

The size of particles directly influences the immunological response elicited, though it is still debatable. Liposomes differ in size and number of lipid layers, ranging from 0.025 to 2.5 μm.^98^ Liposomes used in vaccinations have been displayed to make a protection against TB and were nanoscale (>1 μm).^52^, ^99^ After intravenous *M. tb* infection, vaccines containing microstructured liposomes and HspX are reported to minimize lung inflammation while preserving lung function and structure. Due to their ability to elicit specific immune responses and their microstructured nature, microstructured liposomes are efficient antigen carriers.^89^ However, the combination of adjuvant with liposome, not the liposomes alone, plays a key role in the evoked immunological response. By using an HSPX subunit vaccine in combination with BCG in a prime‐boost strategy, immunity to TB might be enhanced even if BCG is used as the prime vaccine and microstructured liposomes are used as the booster.^100^, ^101^

TLRs, based on their cellular location and the ligands that they bind in pathogen‐associated molecular patterns, are categorized into two types. TLR3 detects polyinosinic‐polycytidylic acid, a compound mimicking viral infection that triggers antiviral responses by increasing IFN‐γ signaling and inflammatory cytokines.^102^ When TLR3 agonists were combined with DDA, antigen‐specific CD8+ T cells produced IFN‐γ, TNF‐α, and IL‐2 and were more likely to cross‐present antigens on class I MHC molecules.^103^ DDA liposomes were found to be more effective when containing BCG polysaccharide nucleic acid (BCG‐PSN). In BCG‐PSN, polysaccharides and nucleic acids (like CpG) are present; it was obtained by hot phenol extraction from *M. bovis bacillus Calmette‐Guerin*.^104^ Through the TLR9 pathway, B cells and plasmacytoid dendritic cells can be directly activated by CpG motifs (CpG ODN).^105^ Mycobacterial cell walls contain TDM, which stimulates the immune system. CAF01 is a TDM analog comprising DDA. TDB has been displayed to stimulate Th1‐type cellular immunological responses.^106^ The emulsion and liposomal adjuvants both protected against a mycobacterial challenge in preclinical studies, but the liposomes‐based (AS01) induced a stronger immune response. This was determined by a stronger IFN‐γ response, as well as a contribution from CD8+ T cells. However, the mechanisms underlying the responses remain largely unknown.^107^ In human volunteers, this hierarchy was observed, and AS01 was selected as the preferable adjuvant candidate for the vaccination antigen M72.^92^

## 6 CONCLUSION

In comparison to any other pathogen, TB kills more people than any other and more than ever, it is urgent that a universally effective vaccine be developed. A reliable vaccine is a must to achieve the WHO targets set for the End TB Strategy. In animal models and clinical studies, TB vaccine candidates have indicated safety, immunogenicity, and effectiveness. Compared to BCG vaccines, TB vaccines are comparable or even more promising. Currently, TB vaccines are being tested with four adjuvants. While our knowledge about the mechanisms of action of these adjuvants is improving, they were established during a time when IFN‐γ was the dominant screening method. New technical progress in vaccine research, for example, single B/T cell whole transcriptome analysis and systems immunology, results in significant discoveries. Therefore, examining the intersections between innate and adaptive immunity is essential. One of the variables that will be critical in the development of an effective vaccine is the participation of B‐cells and antibodies. The discovery of downregulated invariant natural killer T cells in the blood of TB patients exhibits those antibodies that could be employed to target latent infection. Moreover, the activation of these cells through galactosylceramide could destroy latently infected cells. In some species, vaccination with liposomal vaccines may provide prolonged protection against *M. tb* infection. Considering these data, it appears that a liposomal adjuvant system is excellent for vaccination against TB and other intracellular infections, as well as tumors. Systematic analyses of clinical trials can contribute to achieving important information on developing new TB adjuvants and enhancing the effect of adjuvants in next‐generation TB vaccines.

## AUTHOR CONTRIBUTION

**Melika Moradi**: Data curation; writing and drafting. **Farzaneh Vahedi**: Data curation; writing and drafting. **Mohammad Sholeh**: Data curation; writing—original draft. **Mortaza Taheri‐Anganeh**: Conceptualization; writing—review and editing. **Zahra Dargahi**: Formal analysis; writing—review and editing. **Roya Ghanavati**: Data curation; writing—review and editing. **Seyyed Hossein Khatami**: Conceptualization; writing—review and editing. **Ahmad Movahedpour**: Funding acquisition; supervision; writing—review and editing.

## CONFLICT OF INTEREST STATEMENT

The authors declare no conflict of interest.
